# Lifting of the 1,000 wheat exome project SNPs from *Triticum aestivum* cv. Chinese Spring assembly RefSeq v1.0 to RefSeq v2.1

**DOI:** 10.1186/s13104-023-06496-8

**Published:** 2023-09-14

**Authors:** Akshaya Vasudevan, Sylvie Cloutier

**Affiliations:** 1 Agriculture and Agri-Food Canada, Ottawa Research and Development Centre, Ottawa, ON K1A 0C6 Canada; 2 Department of Biology, University of Ottawa, Ottawa, ON K1N 6N5 Canada

**Keywords:** Liftoff, SNP lifting, RefSeq v2.1, Chinese Spring reference genome

## Abstract

**Objective:**

The 1,000 wheat exome project captured the single nucleotide variants in the coding regions of a diverse set of 890 wheat accessions to analyse the contribution of introgression to adaptation of wheat. However, this highly useful single nucleotide polymorphism (SNP) dataset is based on RefSeq v1.0 of the International Wheat Genome Sequencing Consortium (IWGSC) assembly of the bread wheat genome of Chinese Spring. This reference sequence has recently been updated using optical maps and long-read sequencing to produce the improved RefSeq v2.1. Our objective was to develop a reliable high-density SNP dataset positioned onto RefSeq v2.1 because it is the current standard reference sequence used by wheat researchers.

**Results:**

The 3,039,822 SNPs originally positioned on RefSeq v1.0 were projected to v2.1 using Liftoff with four different flanking regions, and 2,946,536 SNPs were consistently lifted to the same location irrespective of the flanking region lengths. Of these, 2,799,166 were located on the ‘+’ ve strand. The distribution of the SNPs across the 21 chromosomes on RefSeq v2.1 was similar to that of RefSeq v1.0. Among the SNPs that were based on unanchored scaffolds in RefSeq v1.0, 11,938 were projected to one of the 21 pseudomolecules in the upgraded assembly. This SNP dataset constitutes a much-needed standardized resource for the wheat research community.

**Supplementary Information:**

The online version contains supplementary material available at 10.1186/s13104-023-06496-8.

## Introduction

As of January 2021, reference sequences had been generated for 798 land plants [1]. With the advances in sequencing technologies, high-quality genome assemblies of polyploid species are now more readily produced [1]. Availability of gold-standard reference genomes is important for application in crop improvement through characterization of genomic regions underlying traits of interest and for the utilization of genetic diversity from wild relatives [2]. Wheat (*Triticum aestivum* L.) is an allohexaploid species with a genome estimated at ~ 16 Gb that contains the A, B and D subgenomes [3]. The first assembly of bread wheat cultivar Chinese Spring, based on chromosome survey sequences and released in 2014 [4], provided the baseline for the subsequent iterations. The requirement for a highly contiguous and well annotated genome eventually led to the development of the first pseudomolecule-level assembly of Chinese Spring (RefSeq v1.0) totalling 14.5 Gb, a feat that was accomplished by the International Wheat Genome Sequencing Consortium (IWGSC) using Illumina sequencing and DeNovo MAGIC assembly [5]. Using optical map and PacBio long-read sequencing data, the assembly quality was further improved to produce IWGSC RefSeq v2.1, in which multiple scaffolds were anchored, reoriented and relocated [6]. Consequently, the quality of the annotation was improved, and the number of high confidence genes in the annotation also increased from 105,534 genes in v1.1 to 106,913 in v2.1 [6].

The current day hexaploid wheat evolved ~8000 years ago through chance hybridizations between domesticated emmer wheat (*Triticum turgidum* L. ssp. *dicoccum*) and goatgrass (*Aegilops tauschii* Coss.) [7, 8]. The limited polyploidization events, the subsequent domestication and breeding have restricted the genetic diversity in hexaploid wheat, especially in the D subgenome. Considering the need to understand gene flow from progenitors into hexaploid wheat and their role(s) in adaptation and evolution, an extensive 1,000 wheat exome project was designed and executed through an international collaboration [9]. Here a diverse panel of 890 accessions comprised of hexaploid wheat cultivars, landraces and wild and domesticated tetraploid progenitors were taken up and subjected to exome capture and sequencing. Using the IWGSC RefSeq v1.0 as reference, single nucleotide polymorphisms (SNPs) were called, a haplotype map was generated, and the preponderance of introgressions in wheat adaptation was investigated by He et al. [9]. This publicly available SNP dataset is an important genomic resource to the international wheat research community. However, it is based on v1.0 which is no longer the standard reference genome for wheat research. To ensure its continued utility, projection of this extensive SNP dataset unto RefSeq v2.1 is urgently needed. Here we outline a strategy based on the repurposing of Liftoff [10] and present the process and outcome of the lifting-over of the 1,000 wheat exome SNP dataset to the IWGSC RefSeq v2.1 assembly.

## Methods

**Fig. 1:**
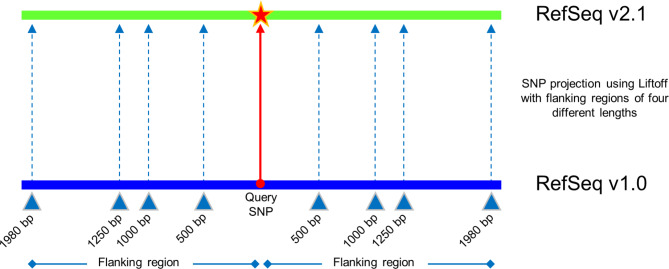
Strategy implemented for projecting SNPs from IWGSC RefSeq v1.0 to RefSeq v2.1 using Liftoff

The Liftoff tool was originally developed for projecting annotations from one reference genome assembly to another without the use of chain files [10]. For this purpose, features are assigned in a hierarchy, for instance, gene as parent feature and exon as child feature. The parent feature (gene sequence) is aligned to the target, following which, the location of the child feature is deduced. Here we repurposed Liftoff for lifting-over SNPs, by assigning the SNP and the corresponding flanking regions of a chosen length as the child and parent features, respectively. In this process, the flanking regions of each SNP are aligned to the target genome and the “region” between them delineates the single nucleotide position of the SNP (Fig. 1). To project the SNPs from the 1,000 exome project, the variant call format (VCF) file was downloaded from the Wheat URGI repository (https://wheat-urgi.versailles.inra.fr/Seq-Repository/Variations). The 3,039,822 SNPs in the VCF file were split into 16 parts for ease of computational analyses, and the result files were merged after processing. The VCF files were converted to GFF files using a custom R script. For each SNP, the GFF file contained the parent feature, i.e., the flanking region start and end coordinates, and the child feature, i.e., the SNP coordinates per se. Each SNP and the flanking regions were assigned an ID, which associated the SNPs with their corresponding flanking regions. Strand orientation was not included in the 1,000 wheat exome VCF file. Since providing the plus strand alleles is the standard convention for publicly available SNP datasets [11], the strand orientation for all SNPs in the input was labeled as ‘+’.

A flanking region of 1,980 bp on either side of the SNP was initially used because the average transcript length in the annotation RefSeq v1.1 was 3,961 bp; hence, this length can be argued to represent the average length of conserved regions in the genome. To test the ability of Liftoff to project the SNPs from v1.0 to v2.1, the following flanking region lengths were also assessed: 500 bp, 1,000 bp and 1,250 bp, and only the consensus set of SNPs lifted to the same location with all four flanking region lengths was retained. Liftoff uses Minimap2 [12] for sequence alignment which requires sequences ≥ 100 bp; hence, all flanking region lengths tested met the criteria. Further, the chromosome-to-chromosome lift-over option was not used in Liftoff to allow SNPs to be projected onto different chromosomes or onto newly anchored scaffolds of RefSeq v2.1.

The new v2.1 SNP coordinates corresponding to the v1.0 SNPs of the 1,000 exome VCF file were assigned using their corresponding SNP IDs. The preparation of input files, merging and formatting of files and output summarization steps were performed using custom R scripts and bash. The functional annotation of the SNPs was performed using IWGSC RefSeq v2.1 on SnpEff [13]. The database construction was carried out using both low- and high-confidence gene models from annotation v2.1 using the build option in SnpEff. With the VCF file as input, functional annotation was performed using the eff option with default parameters.

## Results

The number of SNPs lifted-over using the four flanking region lengths is summarized in Supplementary Tables 1–4. The number of SNPs lifted-over ranged from 3,017,212 with the longest flanking regions of 1,980 bp to 3,020,764 with the shortest flanking regions of 500 bp. Comparisons across all GFF3 output files revealed that 2,946,536 SNPs were consistently projected to the same location regardless of the length of the flanking regions. Of these, 2,799,166 (95%) were located on the ‘+’ ve strand (reference genome orientation), while 147,370 (5%) were in the ‘-’ ve strand. In summary, 2,799,166 of the 3,039,822 SNPs (92%) of the 1,000 wheat exome project were lifted-over from RefSeq v1.0 to RefSeq v2.1.

**Fig. 2:**
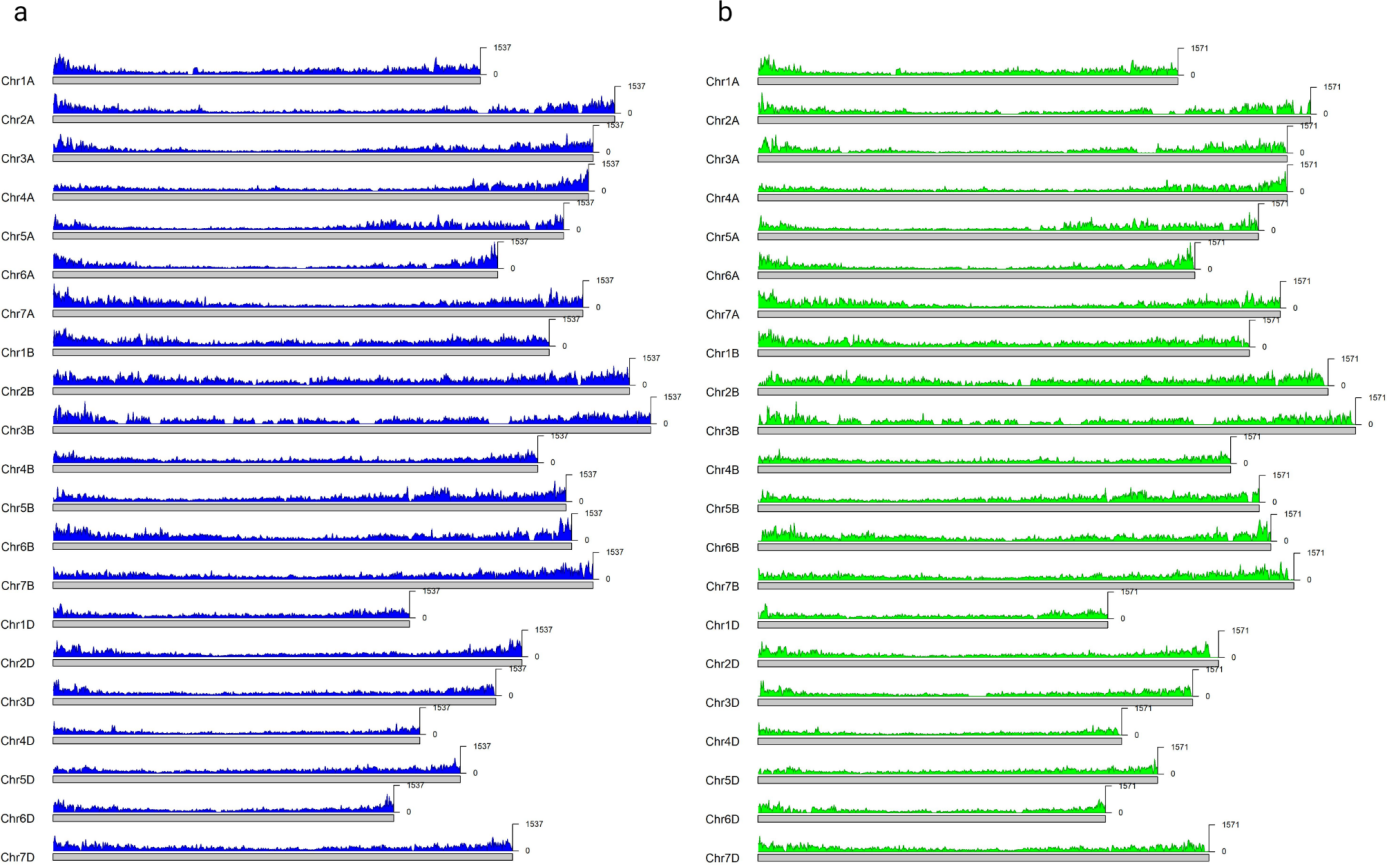
SNP density plots. **(a)** Distribution of 3,039,822 SNPs on IWGSC RefSeq v1.0; **(b)** Distribution of 2,799,166 SNPs on IWGSC RefSeq v2.1

A total of 35,284 out of the 39,822 SNPs originally positioned on the unanchored scaffolds (chrUn) in RefSeq v1.0 were lifted to the same loci regardless of the flanking region lengths. Of those, 11,938 were assigned to a position and projected onto one of the 21 assembled chromosomes. The vast majority of the SNPs were lifted to the same location in both versions, but 5,949 SNPs ended up projected to a different chromosome from v1.0 to v2.1 (Supplementary Table 5). This did not however affect the overall distribution pattern of SNPs across the 21 chromosomes which remained similar between RefSeq v1.0 and v2.1, with higher density of SNPs in the distal and interstitial regions of the chromosomes (Fig. 2).

When the functional annotation of SNPs based on RefSeq v2.1 was carried out, ~ 5.4 million effects were predicted for the ~ 2.8 million SNPs. Effects are defined as the potential consequences of the variants on transcription and translation. For instance, a SNP in a genic region might be present in either exons or introns in differentially spliced isoforms, which in turn will influence the outcome of the variant. Hence, the number of effects determined by the snpEff tool is higher than the number of SNPs. In the annotation by He et al. [9] of the variants based on RefSeq v1.0 annotation, 1.4% of the SNPs were possibly deleterious, including splice site changes and start/stop gained or lost variants, and 10.5% of them were non-synonymous. Based on the RefSeq v2.1-dependent functional annotation, 1.5% and 8.7% of the SNPs were potentially deleterious and non-synonymous, respectively. With regards to the multiple effects of the SNPs lifted to RefSeq v2.1, the majority of the effects (89%) were predicted to have no impact on the proteome because they were either non-coding variants or associated with non-coding genes, as per the SnpEff categorization. A total of 4.7% of the effects were low impact such as synonymous variants, 5.8% were moderate impact with a possibility of affecting the encoded proteins, and 0.4% were high impact with significant effect on the coded proteins. Based on the functional classes, 58.6% of the effects were categorized as missense and 40.5% as silent. The number of deleterious ‘start lost’, ‘stop gained’ and ‘stop lost’ effects were 499, 15,176 and 1,583, respectively. In reference to genic regions, most SNPs were located in intergenic regions, followed by those located in the downstream and upstream regions. However, ~ 20% of the SNP effects were in the intronic and exonic regions of genes and 0.5% were localized at the splice site junctions (Table 1).

**Table 1:** Percent SNP effects based on their location respective to the nearest transcript

Location	Count	Percentage
Downstream	1,240,165	22.77
Exon	557,842	10.24
Intergenic	1,887,968	34.66
Intron	594,000	10.90
Splice site acceptor	2,012	00.04
Splice site donor	1,901	00.04
Splice site region	28,984	00.53
Upstream	1,035,877	19.02
3’-UTR	70,492	01.29
5’-UTR	28,030	00.52

## Discussion

Chain files are required for the widely-used VCF and annotation lift-over tools such as UCSC LiftOver [14], CrossMap [15] and Picard’s LiftOverVcf [16]. Chain files encompass the pairwise alignment information of two sequences capturing the homologous blocks between genomes under comparison. Generating a chain file for large and complex genomes such as wheat is computationally intensive. Here we explored the repurposing of the Liftoff tool, originally designed for projecting coding sequences onto genome, to project millions of SNPs from the Chinese Spring RefSeq v1.0 onto the latest v2.1. Liftoff does not use the computationally intensive chain files. In this repurposing exercise, SNPs were defined as child feature, and SNP flanking regions of different lengths were concurrently used as parent features. Extracting and utilising only the SNPs lifted to the same loci consistently, regardless of the flanking region lengths, ensured that only the most reliable SNP coordinates were retained in the final dataset projected onto RefSeq v2.1.

Optical map and PacBio long-read sequencing were used to refine the Chinese Spring reference assembly and produce RefSeq v2.1 [6]. The utilization of an optical map led to the anchoring of 279 previously unanchored scaffolds, the orientation correction of 354 scaffolds and the relocation of 233 scaffolds [6]. This improved RefSeq v2.1 assembly validated 90% of the IWGSC RefSeq v1.0. As such, it was not unexpected to find that more than 99% of the SNPs were projected to the homologous position across the two assembly versions. The lifting of 11,938 SNPs from ChrUn in RefSeq v1.0 to one of the 21 assembled chromosomes is likely the outcome of the anchoring of previously unanchored scaffolds. A proportion of this lifting, such as lifting from Chr1A (v1.0) to Chr5A (v2.1), Chr2D (v1.0) to Chr4A (v2.1) etc., can be attributed to the relocation of scaffolds to other chromosomes across assemblies. In addition, during the assembly improvement process, small scaffolds were discarded [6], which could have led to the lift-over of SNPs from these scaffolds to a highly homologous region in other chromosomes or onto ChrUn, or these could have remained unprojected. The lifting of 98% of genes from RefSeq v1.0 to RefSeq v2.1 with high accuracy was achieved only because Insertion Site-Based Polymorphisms (ISBP)-dependent complexity reduction strategy was utilized [6]. The fact that the whole RefSeq v2.1 was used as the target might also be the reason for incorrect anchoring of a small set of SNPs. Because of its superior quality, RefSeq v2.1 is now the preferred reference genome assembly version of the wheat community. The use of the SNP variants from v1.0 and their projection onto v2.1 on an ad hoc basis is impractical, time-consuming and could easily lead to errors. It was therefore urgent and crucial to project all SNPs onto RefSeq v2.1 to provide this valuable resource for the whole wheat research community. In conclusion, we successfully lifted the 1,000 wheat exome SNP data unto the IWGSC RefSeq v2.1. The public availability of this standardized genome-wide dataset is expected to spur its continued utilization by the wheat research and breeding communities by ensuring consistency and reliability and, in doing so, to contribute to accelerating discoveries.

### Data Availability

The VCF file with the SNPs projected onto IWGSC RefeSeq v2.1 has been deposited in Zenodo (DOI: 10.5281/zenodo.7852690).

### Electronic supplementary material

Below is the link to the electronic supplementary material.

Supplementary Material 1

## Data Availability

The VCF file with the SNPs projected onto IWGSC RefeSeq v2.1 has been deposited in Zenodo (DOI: 10.5281/zenodo.7852690).

## References

[1] Marks RA, Hotaling S, Frandsen PB, VanBuren R (2021). Representation and participation across 20 years of plant genome sequencing. Nat Plants.

[2] Bevan MW, Uauy C, Wulff BBH, Zhou J, Krasileva K, Clark MD (2017). Genomic innovation for crop improvement. Nature.

[3] Arumuganathan K, Earle ED (1991). Nuclear DNA content of some important plant species. Plant Mol Biology Report.

[4] IWGSC (2014). A chromosome-based draft sequence of the hexaploid bread wheat (*Triticum aestivum*) genome. Science.

[5] IWGSC (2018). Shifting the limits in wheat research and breeding using a fully annotated reference genome. Science.

[6] Zhu T, Wang L, Rimbert H, Rodriguez JC, Deal KR, De Oliveira R, Choulet F, Keeble-Gagnère G, Tibbits J, Rogers J (2021). Optical maps refine the bread wheat Triticum aestivum cv. Chinese Spring genome assembly. Plant J.

[7] Feuillet C, Langridge P, Waugh R (2008). Cereal breeding takes a walk on the wild side. Trends Genet.

[8] Huang S, Sirikhachornkit A, Su X, Faris J, Gill B, Haselkorn R, Gornicki P (2002). Genes encoding plastid acetyl-CoA carboxylase and 3-phosphoglycerate kinase of the Triticum/ Aegilops complex and the evolutionary history of polyploid wheat. Proc Natl Acad Sci.

[9] He F, Pasam R, Shi F, Kant S, Keeble-Gagnere G, Kay P, Forrest K, Fritz A, Hucl P, Wiebe K (2019). Exome sequencing highlights the role of wild-relative introgression in shaping the adaptive landscape of the wheat genome. Nat Genet.

[10] Shumate A, Salzberg SL (2021). Liftoff: accurate mapping of gene annotations. Bioinformatics.

[11] Nelson SC, Doheny KF, Laurie CC, Mirel DB (2012). Is ‘forward’ the same as ‘plus’?… and other adventures in SNP allele nomenclature. Trends Genet.

[12] Li H (2018). Minimap2: pairwise alignment for nucleotide sequences. Bioinformatics.

[13] Cingolani P, Platts A, Wang LL, Coon M, Nguyen T, Wang L, Land SJ, Lu X, Ruden DM (2012). A program for annotating and predicting the effects of single nucleotide polymorphisms, SnpEff. Fly.

[14] Hinrichs AS, Karolchik D, Baertsch R, Barber GP, Bejerano G, Clawson H, Diekhans M, Furey TS, Harte RA, Hsu F (2006). The UCSC Genome Browser Database: update 2006. Nucleic Acids Res.

[15] Zhao H, Sun Z, Wang J, Huang H, Kocher J-P, Wang L (2013). CrossMap: a versatile tool for coordinate conversion between genome assemblies. Bioinformatics.

[16] http://broadinstitute.github.io/picard/.

